# National outbreak of Shiga toxin-producing *Escherichia coli* O157:H7 linked to mixed salad leaves, United Kingdom, 2016

**DOI:** 10.2807/1560-7917.ES.2018.23.18.17-00197

**Published:** 2018-05-03

**Authors:** Maya Gobin, Jeremy Hawker, Paul Cleary, Thomas Inns, Daniel Gardiner, Amy Mikhail, Jacquelyn McCormick, Richard Elson, Derren Ready, Tim Dallman, Iain Roddick, Ian Hall, Caroline Willis, Paul Crook, Gauri Godbole, Drazenka Tubin-Delic, Isabel Oliver

**Affiliations:** 1 Field Epidemiology Services, Public Health England, London, United Kingdom; 2 NIHR Health Protection Research Unit in Gastrointestinal Infections, University of Liverpool, Liverpool, United Kingdom; 3 Field Epidemiology Training Programme, Public Health England, London, United Kingdom; 4 Centre for Infectious Disease Surveillance and Control, Public Health England, London, United Kingdom; 5 Emergency Response Department Science and Technology, Public Health England, Salisbury, United Kingdom; 6 Food Water and Environmental Microbiology Laboratory Porton, Public Health England, Salisbury, United Kingdom; 7 Incidents and Resilience Unit, Food Standards Agency, London, United Kingdom; 8 NIHR Health Protection Research Unit in Evaluation of Interventions at the University of Bristol, Bristol, England

**Keywords:** Salad leaves, Whole genome sequencing, Escherichia coli O157, Shiga toxin-producing E. coli - STEC, outbreaks

## Abstract

We investigated a large outbreak of *Escherichia coli* O157 in the United Kingdom (UK) with 165 cases between 31 May and 29 July 2016. No linked cases were reported in other countries. Cases were predominately female (n = 128) and adult (n = 150), 66 attended hospital and nine had features of haemorrhagic uraemic syndrome. A series of epidemiological studies (case–control, case–case, ingredients-based and venue-based studies) and supply chain investigations implicated mixed salad leaves from Supplier A as the likely outbreak vehicle. Whole genome sequencing (WGS) indicated a link with strains from the Mediterranean and informed the outbreak control team to request that Supplier A cease distributing salad leaves imported from Italy. Microbiological tests of samples of salad leaves from Supplier A were negative. We were unable to confirm the source of contamination or the contaminated constituent leaf although our evidence pointed to red batavia received from Italy as the most likely vehicle. Variations in Shiga toxin-producing *E.* *coli* surveillance and diagnosis may have prevented detection of cases outside the UK and highlights a need for greater standardisation. WGS was useful in targeting investigations, but greater coverage across Europe is needed to maximise its potential.

## Introduction

In June and July 2016, a large outbreak of Shiga toxin-producing *Escherichia coli* (STEC) serotype O157:H7 occurred in the United Kingdom (UK). The increase was first observed in the south-west of England where isolates of STEC O157:H7 phage type (PT) *34 eae+* *stx*2+ *stx*1− were recovered from 24 cases reporting gastrointestinal symptoms within 1 week (20 to 26 June 2016). This represented a 10-fold increase over the expected rate at this time of year in England and Wales. Whole genome sequencing (WGS) revealed that the isolates belonged to a 5-SNP single linkage cluster designated: 5.156.1329.2502.2965.3081.%.

**Figure 1:**
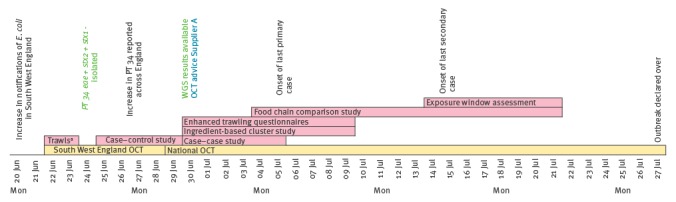
Timeline of investigation, Shiga toxin-producing *Escherichia coli* outbreak, United Kingdom, June–July 2016

OCT: outbreak control team; WGS: whole genome sequencing.

^a^ Standard trawling questionnaire.

A local outbreak investigation was initiated on 22 June 2016 (Figure 1). Epidemiological and environmental investigations indicated a possible link to salad vegetables consumed at catering premises (e.g. restaurants or cafés). Cases were subsequently observed across England and Wales, and Public Health England (PHE) convened a national outbreak control team (OCT) on 29 June to coordinate the investigation and response.

Pink: epidemiological investigations; yellow: coordination and management of the outbreak investigation; text in green: microbiological investigations; text in blue: control measures.

We describe here the epidemiological, environmental and microbiological investigations of this national outbreak.

## Methods

### Case definitions

Confirmed cases were cases with onset or specimen date from 31 May 2016 onwards and a cultured STEC O157 isolate confirmed at the PHE Gastrointestinal Bacterial Reference Unit (GBRU) as PT 34 *eae+ stx2+ stx1−*, and a member of the 5-SNP single linkage cluster with the address 5.156.1329.2502.2965.3081.%. 

Probable cases were cases with onset or specimen date from 31 May 2016 onwards who either had a reference laboratory-confirmed infection with PT *34 eae+ stx2+ stx1−*, in the absence of WGS results, or had serological evidence of STEC O157 infection and were epidemiologically linked (close or household contacts) to a confirmed case.

Secondary cases were confirmed or probable cases with onset 2 days or more after household contact with another confirmed or probable case.

### Clinical microbiology

Isolates of non-sorbitol-fermenting *E. coli* O157 identified in frontline hospital laboratories, were sent to the GBRU for confirmation, *stx1*, *stx2* and *eae* gene detection, phage typing and WGS [1,2]. Genomes were compared with the sequences held in the PHE STEC O157 WGS database which comprises genomes from more than 2,000 cultures (human and animal isolates) of STEC O157 submitted to GBRU between 1982 and 2015. Frontline laboratories were also requested to send faecal or serum samples to GBRU from symptomatic contacts (diarrhoea (often bloody) and/or abdominal cramps or haemolytic uraemic syndrome (HUS)) of confirmed outbreak cases if conventional culture methods failed to isolate this pathogen.

### Epidemiological investigation

#### Initial hypothesis generation

Detailed questionnaires about exposures were completed by telephone interview for 12 probable cases (all subsequently confirmed) who had onset dates in mid-June and were resident in South West England. This generated the initial null hypotheses that illness was not associated with eating salad leaves (e.g. lettuce, mixed leaves, watercress and/or baby spinach) or consumption of salad leaves at catering premises.

We developed a bespoke web-based questionnaire on consumption of salad vegetables, herbs and fruit to identify any additional hypotheses for investigation and administered it to 75 probable and confirmed cases in England up to 8 July. The results were consistent with the initial null hypotheses.

#### Refined hypothesis generation

The hypotheses were refined to bagged mixed salad leaves supplied by UK producer-supplier Supplier A following epidemiological and supply chain investigations. Four epidemiological studies were undertaken to test the hypothesis with some cases included in more than one study.

#### Case–control study

A case–control study was conducted to test the initial null hypotheses. This study preceded WGS results and included probable cases with onset of illness after 1 June 2016 resident in southern England (most cases at the time of the study were residents in southern England). Controls were aged 18 years and over (all cases at time of study were 18 years or older) and frequency matched by sex and region of residence. Four controls per case were recruited from a commercial online market research panel [3]. Exposure data were collected via a web-based questionnaire for the 10 days before recruitment (controls) or before onset of symptoms (cases). Variables included in the multivariable analysis were exposures included in the hypotheses, sex and region of residence, as well as variables with odds ratio (OR) > 1, p < 0.20 and with at least 20% of cases exposed in single variable analysis.

#### Case–case study

A case–case study of probable and confirmed cases resident in England, with onset of illness after 1 June 2016 was undertaken to supplement the results of the case–control study. Comparator cases were identified from the STEC national enhanced surveillance database using the following inclusion criteria:

- Sporadic cases (not linked to an outbreak and isolates not part of any WGS cluster at the 5-SNP level or below);
- Cases infected with a different serotype (non-O157) and/or PT (not PT 34) to the outbreak strain;
- Cases who had not travelled outside the UK in the week before symptom onset;
- Cases with symptom onset in May or June of the years 2009 to 2016;
- Cases who were 18 years or older at the time of onset.

Food and environmental exposures for outbreak and comparator cases recorded as free text in the enhanced surveillance questionnaires were condensed to single keywords [4]. Age, sex, region of residence and exposures with OR > 1, lower 95% confidence interval (CI) > 1 and with at least 20% of cases exposed in the single variable analysis were included in the multivariable analysis.

#### Ingredient-based cluster study

Fourteen cases ate at two catering premises (a staff canteen and a café) during their incubation period. Both premises received 22 common salad vegetables ingredients from the same wholesale Distributor B who sourced product from Supplier A.

An ingredient-based case–control study was conducted to test the hypothesis that consumption of salad vegetables supplied by Distributor B was associated with illness. The chefs provided a full list of ingredients for all menu items. Exposure data collected for the two premises were pooled for the ingredients analysis.

Exposures with OR ≥ 2, p < 0.1 and with more than 60% of cases exposed in single variable analysis were included in the multivariable analysis. A detailed description of this study is available here [5]. 

#### Food chain comparison study

A venue-based study was undertaken to test the refined hypothesis, using a matched case–control study design (venue as unit of analysis). The exposure was defined as having received salad leaves from Supplier A in the study period. 

The supplier of salad leaves received in June 2016 was ascertained from case and control venues. The supply chain was traced back until the salad leaf producer or the importer was identified. The analysis used simple conditional logistic regression.

A detailed description of this study is described by Inns et al. in this issue of Eurosurveillance [6]. 

#### Exposure window assessment

To inform trace-back investigations, mathematical modelling was undertaken to estimate the period of exposure based on onset dates of cases and likely incubation periods using non-parametric and parametric back-calculation methods [7,8]. Using reported timescales for each stage of the supply chain, the time period during which the implicated product was despatched from Supplier A was estimated.

### Environmental investigations

#### Supply chain investigation

Supply chain investigation was undertaken by local Environmental Health Officers (EHO) and coordinated by the Food Standards Agency (FSA). The investigations focused on salad vegetables: bagged, loose, whole head and other ready to eat salad vegetables. The FSA (working with food safety authorities in other European Union countries) initiated trace-forward and trace-backward investigations for salad vegetables from implicated suppliers and catering premises.

Information from the trace-back and trace-forward investigations were recorded in an MS Excel tracing template and reviewed by the FSA and PHE. Data were extracted, prepared and analysed using FoodChain-Laboratory (FCL) [9]. FCL calculated tracing scores for venues and products under investigation. Venues or products with higher scores were more likely to be implicated in the outbreak [9].

#### Environmental sampling

The following samples were collected by EHOs and submitted to the PHE Food, Water and Environment laboratories (FWE) for testing for STEC: remnants of salad vegetables consumed by cases during their incubation period and any associated retained packaging as well as environmental swabs, salad leaves and seeds from suppliers, wholesale distributors, retail and catering premises. Environmental samples were transported and analysed according to the ISO and PHE standard methods [10-12].

Growing and/or processing procedures for suppliers and wholesale distributors identified in the supply chain investigation were reviewed. A history of staff illness at salad leaf processing venues was sought. Stool samples were obtained for STEC testing from food handlers who reported gastrointestinal illness during May or June.

### Surveillance for re-emergence of the outbreak

Modelling of supply chain timelines and incubation period distributions indicated that all new cases associated with an introduction of a contaminated product at Supplier A would be identified by PT within 39 days of product arrival to Supplier A (90% within 27 days and 99% within 37 days).

Two methods were developed to support the declaration of the end of the outbreak and facilitate detection of any re-emergence: (i) the number of enhanced STEC surveillance questionnaires received at PHE each day was compared with the number expected using the Farrington exceedance algorithm (modified to account for weekend effects) and alerts generated by the algorithm were reviewed for possible re-emergence of the outbreak [13]. (ii) The proportion of STEC O157 PT 34 cases vs STEC O157 of other PTs was evaluated on a daily basis. Both methods were applied for 5 weeks after the last confirmed case was reported based on the modelled estimates described above.

## Results

### Microbiology

**Figure 2:**
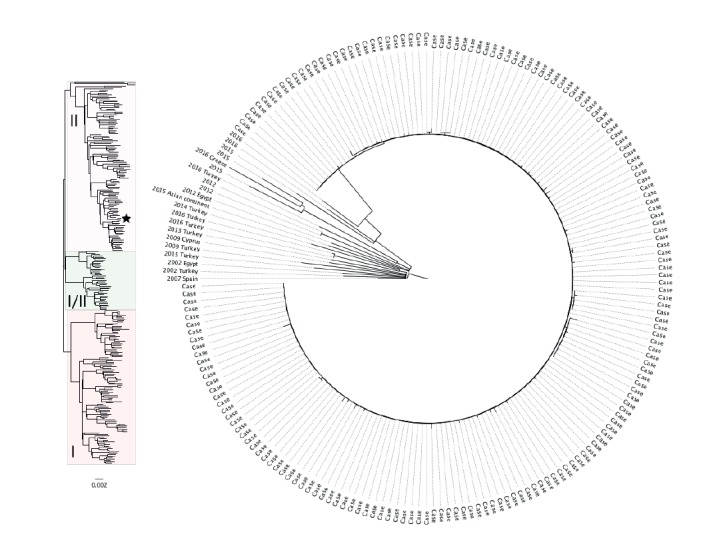
Maximum likelihood phylogenetic trees of the outbreak isolates (n = 165) with nearest genetic neighbours (right panel) and a representative sample of historical isolates of STEC O157: H7 (left panel), shiga toxin-producing *Escherichia coli* outbreak, United Kingdom, June–July 2016

All isolates from 165 confirmed cases belonged to the same 5-SNP cluster (5.156.1329.2502.2965.3081.%), indicating a common source. The clade to which this outbreak cluster belonged was uncommon in the PHE database and was not very diverse, indicative of infrequent sampling from a widespread pool of strains. None of the isolates belonging to this clade were from UK animals. A higher proportion of cases with this clade reported travel to Mediterranean countries, compared with other clades in the database (Figure 2).

PHE: Public Health England.

Left panel: population structure ofSTEC O157:H7 based on 311 representative isolates from the PHE collection with the three major lineages (I, I/II and II) indicated. The position of the outbreak clade is depicted by a black star.

Right panel: the 165 cases from the 5-SNP outbreak cluster and the 22 closest sporadic isolates (same 50-SNP cluster) in the PHE database with country of travel indicated where known. Date is given for non-outbreak cases.

### Epidemiology

**Figure 3:**
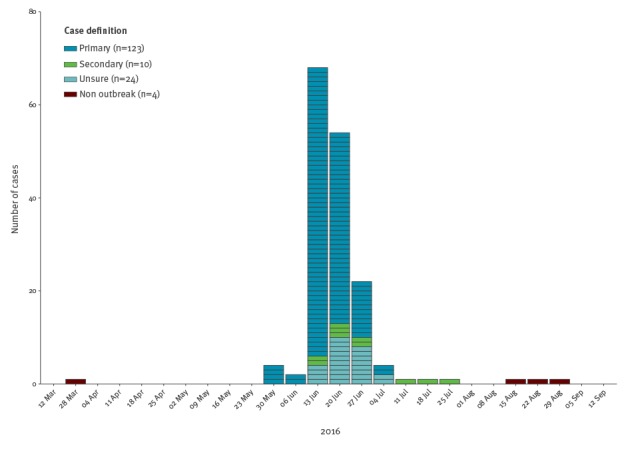
Epicurve of primary, secondary and unsure cases, outbreak of shiga toxin-producing *Escherichia coli*, United Kingdom, 30 May–29 July 2016 (n = 161^a^)

^a^ Onset date unknown for four cases.

There were 124 primary and 10 secondary cases and 31 cases referred to as ‘unsure’ who could not be classified as primary or secondary (Figure 3). They were part of a protracted outbreak/cluster (n = 17), were asymptomatic (n = 2), epidemiologically linked to a confirmed case with a missing date of onset (n = 4), or unsure whether they were a contact of case (n = 8).

The onset dates ranged from 30 May to 29 July 2016. The latest date of onset for a primary case was 5 July. The number of cases peaked between 18 and 20 June 2016. Three cases travelled overseas during their incubation period. Seventy per cent of cases (116/166) were adult women. Cases were between 1 and 98 years-old (median: 51 years; interquartile rang e (IQR): 29–72 years). They were from England (n = 155), Wales (n = 6), Northern Ireland (n = 4) and Scotland (n = 1). Twenty-eight cases were linked to catering premises visited by at least two cases and a further 19 cases were linked to a Care home SE.

Sixty-six cases attended hospital, nine (two of them children) developed HUS and of these, two adult cases died. Among cases with known symptom details (n = 160), 127 cases reported bloody diarrhoea and a further 13 non-bloody diarrhoea.

#### Case–control study

The study included 21 cases and 91 controls. Nineteen cases were female. The median age for cases was 48 years (range: 18–83 years) and 56 years (range: 20–77 years) in controls (p = 0.072). Multivariable analysis using a forward selection procedure showed that illness was associated with mixed salad leaves (OR = 4.56; 95% CI: 1.17–17.79) and salad eaten at one catering premises (OR = 8.30; 95% CI: 1.96–35.15) (Table 1).

**Table 1:** Single variable and final multivariable model, case–control study, outbreak of shiga toxin-producing *Escherichia coli*, United Kingdom, June–July 2016 (n = 112)

Exposure/risk factor	Single variable analysis			Final multivariable model		
	OR	95% CI	p value	aOR	95% CI	p value
Salad in catering premises	18.92	4.69–81.19	< 0.01	8.30	1.96–35.15	<0.01
Mixed salad	8.88	2.75–31.01	< 0.01	4.56	1.17–17.79	0.03
Supermarket SB	3.45	1.14–10.33	0.01	2.70	0.01–0.39	0.12
Any tomato	0.84	0.25–3.33	0.77	1.47	0.29–7.33	0.64
Any lettuce	0.89	0.30–2.90	0.82	1.31	0.34–5.10	0.70
Supermarket SA	2.03	0.60–6.34	0.18	1.21	0.28–5.22	0.79
Region of residence	ND	ND	ND	0.78	0.18–3.43	0.74
Male sex	ND	ND	ND	0.52	0.09–3.19	0.48
Cucumber	0.84	0.29–2.61	0.73	0.41	0.10–1.73	0.22

aOR: adjusted odds ratio; CI: confidence interval; ND: not done; OR: odds ratio.

#### Case–case study

The study included 69 outbreak and 266 comparator cases.

The multivariable analysis indicated an association between outbreak cases and salad consumption (OR = 2.96; 95% CI: 1.62–5.39), eating out at a café (OR = 2.49; 95% CI: 1.26–4.94), consumption of raw vegetables (OR = 2.29; 95% CI: 1.23–4.26) and shopping at Supermarket SC (OR = 2.34; 95% CI: 1.08–5.07) (Table 2).

**Table 2:** Single variable and final multivariable model, case–case study, outbreak of shiga toxin-producing *Escherichia coli*, United Kingdom, June–July 2016 (n = 335)

Exposure/risk factor	Single variable analysis			Final multivariable model		
	OR	95% CI	p value	aOR	95% CI	p value
Male sex	ND	ND	ND	0.61	0.31–1.20	0.15
Age	ND	ND	ND	1.01	0.99–1.03	0.08
Salad	3.11	1.80–5.37	0.01	2.96	1.62–5.39	< 0.01
Eating at a catering premises	2.27	1.24–4.17	<0.01	2.49	1.26–4.94	0.01
Supermarket SC	2.36	1.18–4.72	0.01	2.34	1.08–5.07	0.03
Raw vegetables	3.27	1.84–5.81	<0.01	2.29	1.23–4.26	0.01
Supermarket SD	2.00	1.12–3.56	<0.01	1.83	0.97–3.47	0.06
Salmon	2.89	1.47–5.69	0.01	1.82	0.84–3.94	0.13

aOR: adjusted odds ratio; CI: confidence interval; ND: not done; OR: odds ratio.

#### Ingredient-based cluster study

There were 203 valid responses: 186 respondents ate at the staff canteen, 17 respondents ate at the café. Twenty-four respondents were defined as cases. The median age of cases and non-cases were 51 and 47 years-old, respectively.

Nine salad ingredients met the inclusion criteria for the multivariable analysis, only baby leaf salad was independently associated with illness (OR = 13.15; 95% CI: 1.62–106.50) (Table 3).

**Table 3:** Single variable and final multivariable model for ingredients consumed at the canteen or cafe, outbreak of shiga toxin-producing *Escherichia coli*, United Kingdom, June–July 2016 (n = 203)

Exposure/risk factor	Single variable analysis			Final multivariable model		
	OR	95% CI	p value	aOR	95% CI	p value
Red onion	4.26	1.56–12.32	< 0.01	2.07	0.78–5.50	0.13
Baby mixed leaf	19.71	3.01–822.99	< 0.01	13.15	1.62–106.50	<0.01

aOR: adjusted odds ratio; CI: confidence interval; OR: odds ratio.

A case was defined as a possible, probable or confirmed case.

#### Food chain comparison report

Data were obtained for 86 venues (43 complete case–control venue pairs). The 43 case venues were associated with 57 cases. Thirty case and 10 control venues were supplied with salad leaves by Supplier A.

The odds of a venue being supplied with salad leaves by Supplier A were 7.67 times higher (95% CI: 2.30–25.53) for case venues than for control venues.

#### Exposure window assessment

The back-calculation methods showed that exposure was likely to have occurred throughout June with peak exposure between 10 and 19 June 2016, with an additional likely limited exposure around 27 May 2016. Based on supply chain timelines, this equated to potentially contaminated product arriving to Supplier A between 6 and 14 June 2016.

### Environment

#### Supply chain investigation

The investigation in the south-west of England identified that wholesale Distributor B was linked to 17 catering premises where cases had eaten. Distributor B supplied unwashed mixed baby leaves to these catering premises sourced from three UK producer-suppliers. Produce could not be traced from the catering premises back to a specific producer-supplier because Distributor B did not maintain records of their distribution of incoming batches.

Investigations focused on one of the three UK producer-suppliers, Supplier A, who supplied salad leaves to catering premises linked to cases outside the south west of England. A forward trace could be established from Supplier A to all catering premises linked to two or more cases. Single salad leaf produce purchased by Supplier A for distribution in the UK, was predominantly purchased from wholesale distributors who source produce from other intermediate distributors or directly from farms. Mixing of single leaves occurred at various points along the supply chain before arrival at Supplier A. The investigation was refined to seeds or leaves of non-UK (Mediterranean) origin following analysis of the WGS phylogeny. These included red batavia and rocket grown in Italy and green mizuna and rocket grown in the UK from Italian seeds. Contaminated red batavia received from Italy on 6 June was suspected as the source of the outbreak based on the exposure window assessment and supply chain timelines.

Rapid Alert System for Food and Feed (RASFF) news notification was issued by the UK on 5 July and a request was made by the FSA to the Italian authorities for details of the traceability of potentially implicated salad leaves. Sampling details and information on production at specific premises were also requested.

At least seven Italian suppliers and more than 10 producers across northern and central Italy could have supplied produce to Supplier A for distribution between 6 and 14 June. Italian investigations did not identify any breaches in practice to explain the outbreak. STEC O157 was not detected in 51 samples of salad leaves and vegetables taken from the areas where the suppliers to Supplier A were located. All indicator *E. coli* detected in routine water and salad samples from the implicated farms around the time of the outbreak were within acceptable levels. No detections of STEC O157 were reported in samples from the implicated farms (sampled following the RASFF notification and FSA request).

#### Environmental sampling in the United Kingdom

STEC O157 was not detected in 191 environmental samples: 140 salad items (including rocket, red batavia, mizuna, mixed leaves, cucumber and tomatoes), 37 environmental swabs, nine samples of seeds for salad leaf crops, and five water samples. Samples were obtained from producers and wholesalers (n = 99), catering premises (n = 49), care homes (n = 17), retailers (n = 15) and the homes of cases (n = 11). STEC O128 *stx*2b+*stx*1c+ was detected by PCR and culture in a sample of mixed leaves taken from a fruit and vegetable shop. *Stx* genes were detected by PCR in a sample of red batavia from Supplier A; however, STEC could not be isolated by culture.

Nine samples of leaves taken from a Retailer W, the staff canteen and Care home SE had *E. coli* levels greater than 100 cfu/g (the level used to prompt a review and improvements in production hygiene and quality of the raw product) [14]. No food handlers reported symptoms during May or June.

#### Control measures

On 30 June, following advice from the OCT, Supplier A discontinued using, and advised their customers to withdraw, products containing salad leaves sourced from Italy. This advice remained in place for five weeks.

### Surveillance for re-emergence of the outbreak

The outbreak was declared over on the 27 July 2016, 22 days after the date of onset of the last primary case and 28 days after the control measures taken by Supplier A. Three cases (two primary and one secondary) with isolates within the same 5-SNP single linkage cluster were identified during the 5-week period of enhanced surveillance for re-emergence. Investigation failed to identify a link with the outbreak. Surveillance for re-emergence remained in place for a further 5 weeks after the detection of the three cases. The OCT stood down on 4 October.

## Discussion

Multidisciplinary working, supported by robust, novel and rapid epidemiological studies and extensive supply chain investigations, was vital to identifying the mixed salad leaves from Supplier A as the likely source of the infection in this large national outbreak. WGS provided useful information about the likely origin of the produce and informed the advice from the OCT to Supplier A to discontinue distribution of imported salad leaves.

Contaminated salad leaves have been linked to outbreaks of STEC O157 previously [15]. Contamination with STEC O157 can occur before, during or after harvest [16,17]. STEC O157 can survive on leaf surfaces and within leaf tissue and remain after standard post-harvest decontamination procedures [18,19]. Cut leaves provide a more favourable environment for the organism to survive and proliferate [18]. It is therefore necessary to prevent contamination from the point of growth and harvest through the supply chain to the final processor. Growers, processors and suppliers are required to follow good practice guidelines to control all hazards along the production pathway [20,21].

We were unable to confirm the source of contamination or the constituent leaf that acted as a vehicle for infection, although our evidence pointed to red batavia received from Italy as the most likely vehicle. Our investigation was challenged by the complex international and national supply network for salad leaves which provided multiple opportunities for cross-contamination.

FCL helped manage and visualise delivery data and identified Supplier A as a common link early in the investigation. However, the data available from suppliers, wholesalers and catering premises had different formats and their accounts of how long the products were in the supply chain varied between 2 and 9 days. This limited interpretation at the level of the constituent leaf.

Routine testing practices in the UK and Italy include indicator organisms only (*E. coli* or coliform bacteria) and not STEC O157, which limited our investigation. These methods would not have detected low levels of faecal contamination with sufficient STEC O157 to cause illness [22,23]. In addition, not all routine samples tested were taken during the exposure window. Above average rainfall was recorded in the northern regions of Italy during the exposure window. Transient contamination by surface run-off from neighbouring farmlands would only have been detected if routine testing had coincided with periods of high rainfall [24].

Another challenge we encountered was the lack comparable case information from other European countries. Epidemic Intelligence Information System (EPIS) and Early Warning and Response System (EWRS) alerts did not identify linked cases in other countries, and many countries took more than 10 days to respond. There is no standard approach to subtyping of STEC O157 and most European countries do not undertake WGS. The European Centre for Disease Control (ECDC) currently recommends multilocus variable-number tandem repeat analysis (MLVA) and pulsed-field gel electrophoresis (PFGE), and many countries undertake PFGE only [25]. In England and Wales, isolates of STEC O157 are subjected to phage typing, with WGS introduced for surveillance and outbreak detection in 2015 [1]. PFGE is not directly comparable to phage typing or WGS; therefore, cases associated with this outbreak that may have occurred elsewhere in Europe at the same time would not necessarily have been identified.

There is evidence of the value of WGS in outbreak investigations through the timely and accurate identification of linked cases [26,27]. Phylogenetic analysis may also provide an indication whether the source of contamination is likely to be domestic or imported [28]. In this outbreak, the absence of WGS data from other European countries limited our ability to identify the geographical origin of the contaminated vehicle or evaluate the evolutionary context.

The case definitions used, the nature of the reporting (voluntary or compulsory) and coverage (national or local) of the surveillance schemes for STEC vary between and within countries [29]. In many European countries, surveillance for STEC is regional and on clinical presentation: bloody diarrhoea, HUS or suspected HUS with one national voluntary HUS surveillance scheme [30,31]. HUS was an uncommon presentation in this outbreak and systems reliant on HUS would have failed to identify outbreak cases with diarrhoea only.

Despite these challenges, rapid epidemiological investigations along with supply chain investigations and analysis of WGS data resulted in prompt identification of the likely vehicle and informed the control measures which may have reduced the extent and duration of the outbreak.

Complex supply networks, multiple opportunities for contamination and limited records kept by food businesses highlight the need for robust mechanisms to trace salad leaves from farm to fork. Food businesses and regulators should work together to agree a minimum, high quality dataset for routine use to facilitate product tracing during outbreaks.

The complexity of the supply chain extending across Europe makes European collaboration essential and highlights the need for greater standardisation of surveillance and sharing of information between countries in a timely manner. Further harmonisation of testing across Europe would be beneficial; WGS was useful in targeting our investigations, but greater coverage is needed if its potential is to be maximised. Given the challenges of investigating outbreaks linked to salad items, a combination of epidemiological and other methods undertaken rapidly and in parallel is likely to be needed to identify sources and control outbreaks.
